# A comparison of nest‐site characteristics for two sympatric Estrildid finches (*Uraeginthus* spp.) in Tanzania

**DOI:** 10.1002/ece3.9398

**Published:** 2022-10-08

**Authors:** Nao Ota

**Affiliations:** ^1^ Department of Behavioural Neurobiology Max Planck Institute for Ornithology Pöcking Germany; ^2^ JSPS Overseas Research Fellow Japan Society for the Promotion of Science Tokyo Japan

**Keywords:** blue waxbill, breeding behavior, construction behavior, nest‐building, niche partitioning

## Abstract

It is well known that birds have great diversity in nesting strategies, but we still have limited knowledge of the variation among species that share the habitat. Here, I will report and compare the nesting strategies between the two sympatric songbirds. Blue‐capped (*Uraeginthus cyanocephalus*) and red‐cheeked cordon‐bleus (*Uraeginthus bengalus*) are socially monogamous, biparental songbirds (family Estrildidae) that sympatrically inhabit arid landscapes with trees and bushes in East Africa. Both species build domed nests with grasses that are often located near wasp nests. They also sometimes take over old weaver (family Ploceidae) nests. While these nesting strategies are already described as common behavioral traits in both species in the literature, interspecies variation in these nesting strategies in areas of sympatry is not reported. My initial field observation during their breeding season suggested that whether these nesting strategies were adopted or not varied somewhat between the sympatric cordon‐bleus. Thus, I carried out a more formal investigation to test these differences. I found that red‐cheeked cordon‐bleus built their nests near wasp nests more frequently than blue‐capped cordon‐bleus, while I did not find any other significant differences between the nesting sites of the two species, such as the use of weaver nests, the types of nesting plants, or nest heights. These results suggest that the sympatric cordon‐bleus share several nest‐site characteristics, but that red‐cheeked cordon‐bleus have an affinity for nesting near wasp nests. Further studies will be required to elucidate the costs and benefits of these nesting strategies or the role that adjacency to wasp nests might play in the sympatry of the two species.

## Graphical abstract

This study reports variations in the nesting strategies of two sympatric songbirds, blue‐capped cordon‐bleus and red‐cheeked cordon‐bleus. They are known to build nests near wasp nests or take over old weaver nests, which may contribute to predator avoidance and saving energy in nest building. Although the above two species have been considered to have similar nesting strategies, my study found that red‐cheeked cordon‐bleus build nests near wasp nests more frequently than blue‐capped cordon‐bleus.

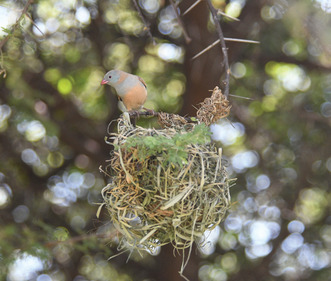

## 1 INTRODUCTION

Birds have evolved various nesting strategies under several selection pressures to increase their reproductive success (Mainwaring et al., 2014). Birds have to select appropriate nest sites and materials in order to minimize predation risk and provide ideal microclimatic conditions for eggs and chicks. For instance, nest sites are shifted to more concealed locations according to the predation risks (Peluc et al., 2008; Schmidt et al., 2006). Nest associations between birds and other species, such as wasps, have been noted for decades in many species (Moreau, 1942; Quinn & Ueta, 2008). Some empirical studies reported that building a nest near wasp nests is beneficial for the reproductive success of birds (Beier & Tungbani, 2006; Joyce, 1993). Some birds are known to take over other weavers' nests, which is assumed to contribute to saving energy for nest building and providing a thermal barrier in cold temperatures (Oschadleus, 2017). On the other hand, such nesting behaviors can cause competitive situations when several species use the same strategy in the shared habitat. To avoid conflicts and interspecific competition, ecologically similar species often use different nest sites (Kosinski & Winiecki, 2004; Vierling et al., 2009).

**FIGURE 1:**
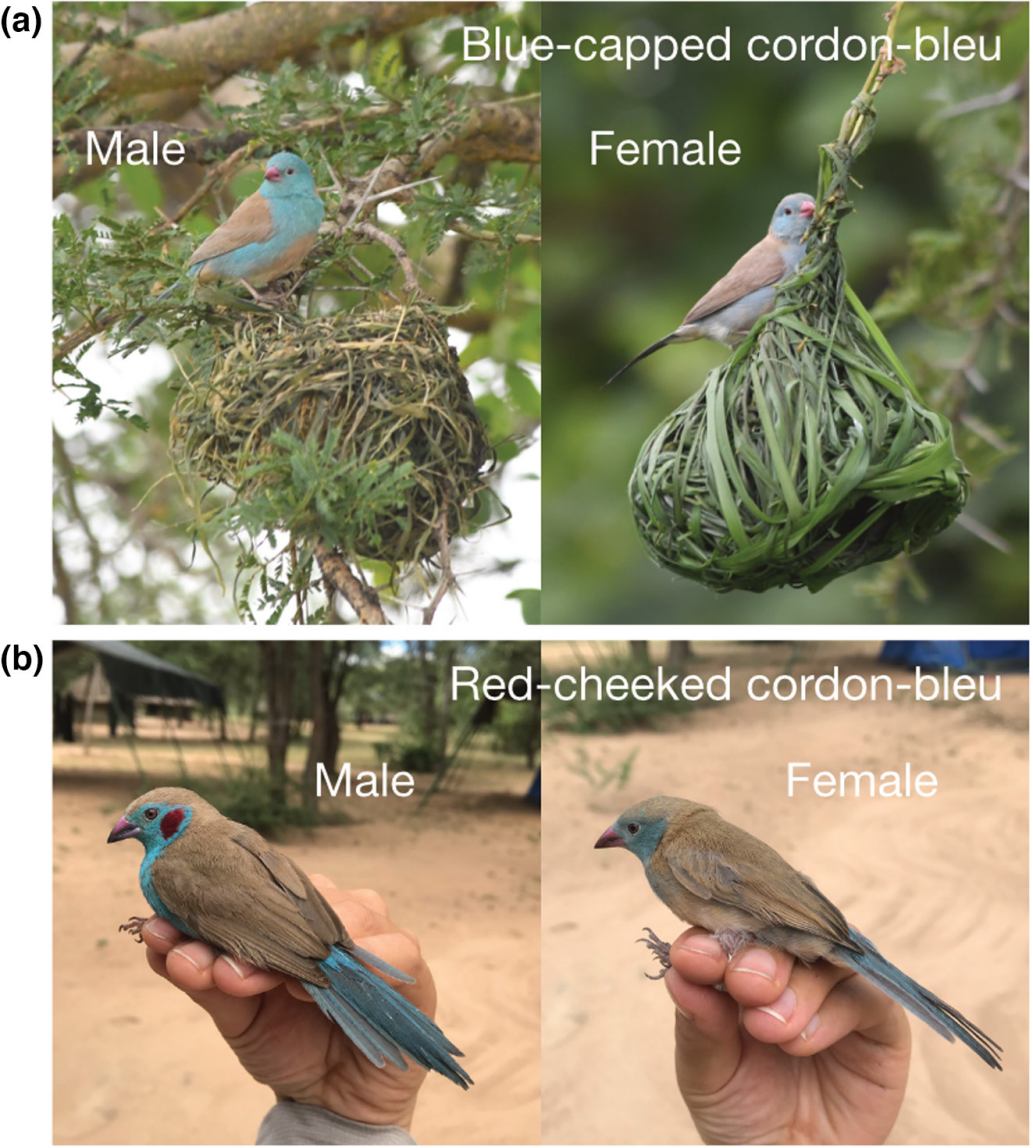
(a) A male (left) and a female (right) blue‐capped cordon‐bleus *Uraeginthus cyanocephalus* perching on weaver nests. (b) A male (left) and a female (right) red‐cheeked cordon‐bleus *Uraeginthus bengalus*. Male blue‐capped and red‐cheeked cordon‐bleus, as their names suggest, have blue caps and red cheeks, respectively. Female blue‐capped cordon‐bleus have b righter blue plumage and pink beak than red‐cheeked.

Blue‐capped cordon‐bleus (*Uraeginthus cyanocephalus*) and red‐cheeked cordon‐bleus (*Uraeginthus bengalus*) are socially monogamous, biparental Estrildid finches from Africa (Figure 1). They are closely related species and have similar life history and breeding behavior, and their habitat overlaps in East Africa (Billerman et al., 2020; Goodwin, 1982; Hockey et al., 2005). Past literature generally mentioned the ecological and behavioral similarities between the two species (Billerman et al., 2020; Goodwin, 1982; Hockey et al., 2005). However, the possibility of adopting different behavioral strategies to avoid conflicts in the shared habitat has been overlooked.

**FIGURE 2:**
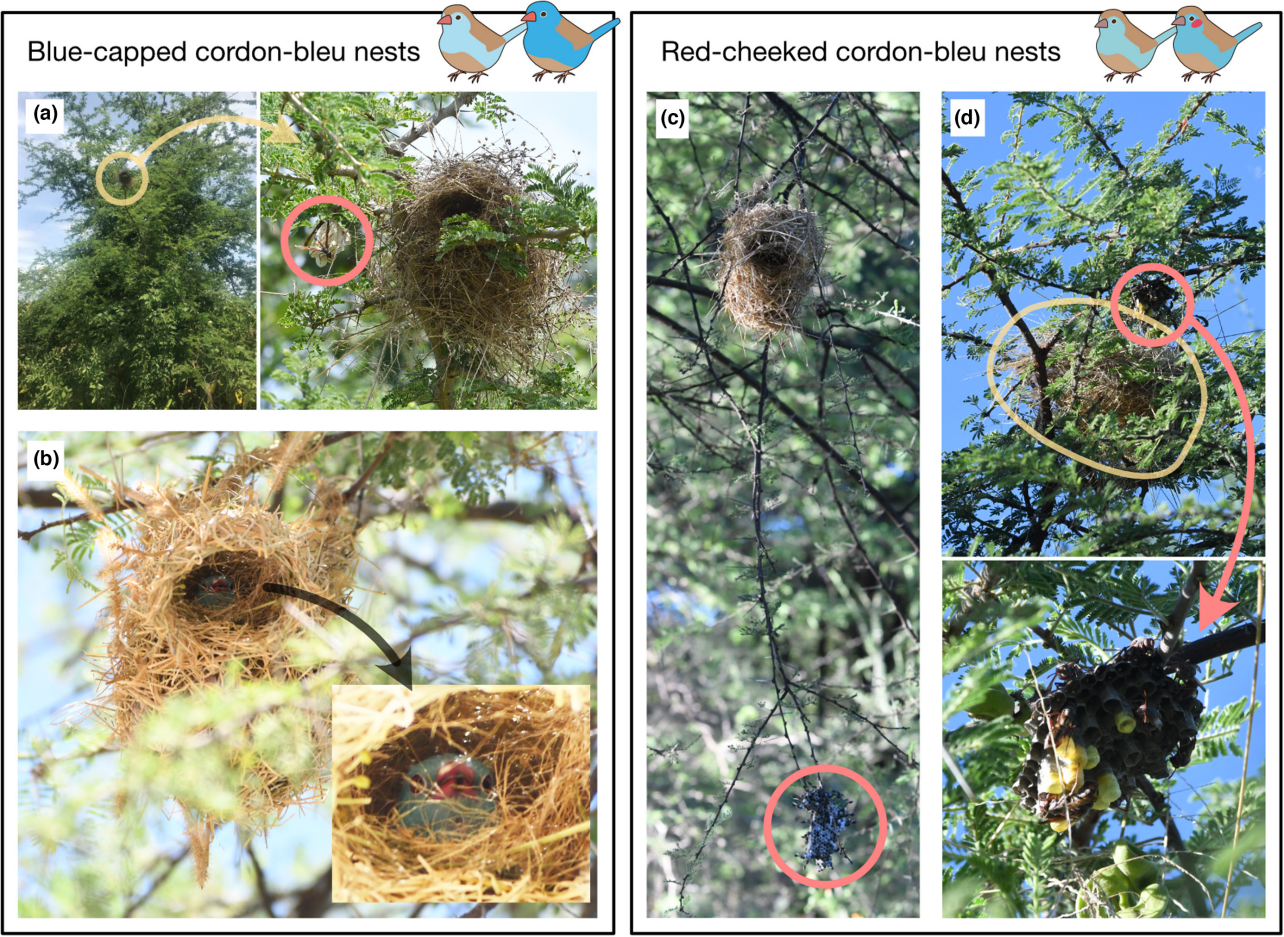
Examples of different types of blue‐capped (a, b) and red‐cheeked (c, d) cordon‐bleus nests examined in this study. Nests were often built on the same branches of wasp nests (a, c, d), high in Acacia, and cordon‐bleus sometimes occupied weaver nests (b, d).

Both blue‐capped and red‐cheeked cordon‐bleus build domed nests in trees mainly using grasses, but sometimes take over weaver (*Ploceus* spp. or *Bubalornis* spp.) old nests (Figure 2b,c). They often build a nest near wasp nests (Hymenoptera: Vespidae) to reduce predation risk (Figure 2a,c,d; Beier & Tungbani, 2006; Billerman et al., 2020; Goodwin, 1982; Hockey et al., 2005). During my fieldwork in Tanzania, I observed that cordon‐bleus used the same nesting strategies as previously reported (Figure 2). During my fieldwork in Tanzania, I observed that cordon‐bleus used the same nesting strategies as previously reported (Figure 2). In addition, I confirmed that two species of cordon‐bleus inhabited and bred sympatrically at my field site, so I expected it would be an ideal opportunity to observe their nesting behaviors and to examine if there are any species differences in the nesting strategies.

I hypothesized that two sympatric cordon‐bleus show different preferences for nest sites, which could lead to avoiding resource competition. If the hypothesis were true, blue‐capped and red‐cheeked cordon‐bleus would build nests in different locations using different resources. I recorded the positions and surrounding conditions of the nests and tested if there were species differences.

## 2 METHODS

### 2.1 Study site

I conducted field observations in Mbeya (GPS coordinates; S 8°43′40″–8°49′06″, E 34°01′50″–34°04′56″) and Iringa region (GPS coordinates; S 7°47′54″–7°49′19″, E 35°05′06″–35°46′04″), Tanzania, during the rainy season from March to May 2019, when cordon‐bleus usually start pairing and breeding (Goodwin, 1982). I observed that both blue‐capped and red‐cheeked cordon‐bleus inhabited all study sites (Ota, personal observation).

### 2.2 Nest observations

I recorded 43 blue‐capped cordon‐bleu nests (41 in Mbeya and two in Iringa) and 15 red‐cheeked cordon‐bleu nests (seven in Mbeya and eight in Iringa). The primary purpose of the expedition was to observe the multimodal courtship display of wild cordon‐bleus (Ota, 2020), but I could also observe nest‐building behavior and the nests during the study (Figure 2; Video S1). When attempting to observe the courtship behavior of cordon‐bleus, I usually focused on individuals holding pieces of nest material in their beaks since cordon‐bleus perform courtship displays by holding a piece of nest material (Ota, 2020; Ota et al., 2015). A cordon‐bleu holding a piece of nest materials often carried it to their nests (Video S1) rather than performing courtship displays. I defined that cordon‐bleus were using the nests when bringing or lining nest materials (Video S1) or staying inside the nest (Figure 2b). When I found a cordon‐bleu nest, I got as close as I could to the nest and recorded its location (longitude and latitude) by BirdLasser, a mobile application to record the observation of birds with the GPS function of the iPhone 7 (Lee & Nel, 2020).

For all nests found, I quantified the following conditions: whether the nest owners are blue‐capped or red‐cheeked cordon‐bleus (Figure 1), if a wasp nest was found on the same perch with nests of cordon‐bleus (Figure 2a,c,d) and if cordon‐bleus built the nest themselves or used an old weaver nest (Figure 2b,c). I also recorded in what types of plants cordon‐bleus nested. Both cordon‐bleu species usually nested on *Acacia* spp. or Acacia‐like trees (*Faidherbia albida*), those are thorny trees with fern‐like leaves and are dominant in their habitat. They also nested on shrubs or broadleaf trees as described in the literature (Goodwin, 1982). I therefore categorized the nest plants as follows: (1) Acacia(−like) trees, (2) shrubs, and (3) broadleaf trees. For a subset of nests (26 blue‐capped nests and 13 red‐cheeked nests), I recorded the nest height by measuring from the ground to the bottom of cordon‐bleu nests following the procedure of a past study (Beier & Tungbani, 2006). I collected nest height data on fewer nests because I decided to collect these data in the middle of my fieldwork, not because of any external measuring constraints (e.g., the nests were too high). Using these data, I examined differences in the nesting strategy between blue‐capped and red‐cheeked cordon‐bleus.

### 2.3 Statistical analyses

To test the species differences in nesting strategies between blue‐capped and red‐cheeked cordon‐bleus, I conducted the following statistical analyses: Fisher's exact test was used to test the species differences in the proportion of the association with wasp nests, the use of weaver nests, and the nested plant types. Student's *t*‐test was used to test the species differences in the nest heights after confirming that the data were normally distributed (Shapiro–Wilk test: *W* = 0.973, *p* = .458). All statistical analyses were performed using R 3.4.4 (R Core Team, 2018). All data used in the analyzes are provided in the supplementary information (Appendix S1).

## 3 RESULTS

**FIGURE 3:**
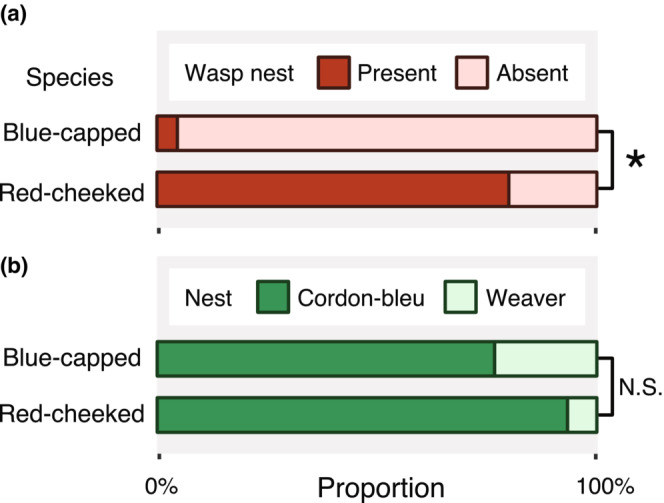
The bars indicate the proportions of (a) nests with (dark red) and without (pink) a wasp nest, and (b) cordon‐bleu nests (dark green) and weaver nests (light green). An asterisk indicates a statistically significant difference (*p* < .05) between species and N.S. nonsignificant.

Red‐cheeked cordon‐bleus built their nests on the same branch as a wasp nest (80%, 12 of 15 nests) more often than blue‐capped cordon‐bleus (4.7%, two of 43 nests; Fisher's exact test, *p* < .001; Figure 3a). There was no significant difference in the use of weaver nests by blue‐capped cordon‐bleus (23.3%, 10 of 43 nests) and red‐cheeked cordon‐bleus (6.7%, one of 15 nests; Fisher's exact test, *p* = .257; Figure 3b). The only weaver nest used by the red‐cheeked cordon‐bleus was accompanied by a wasp nest (Figure 2c). I never observed blue‐capped cordon‐bleus in weaver nests next to wasp nests.

There seemed to be no clear difference between the two cordon‐bleus in their preference for nesting positions. There were no significant species differences in nested plant types and the nest height. Both species mainly nested on the Acacia (‐like) trees (>86%; 39 of 43 nests in blue‐capped, 13 of 15 nests in red‐cheeked) and occasionally on other shrubs (two of 43 nests in blue‐capped, zero of 15 nests in red‐cheeked) and broad‐leaved trees (two of 43 nests in blue‐capped, two of 15 nests in red‐cheeked). There was no significant species difference in the proportion of nested plant types (Fisher's exact test, *p* = .441). The nest height was also not significantly different between species (blue‐capped: median ± SD = 255.5 ± 72.8 cm, red‐cheeked: 250 ± 72.0 cm; Student's *t*‐test: *t* = 0.119, *p* = .906).

## 4 DISCUSSION

The takeover of weaver nests and the association with wasp nests were observed in both blue‐capped and red‐cheeked cordon‐bleus (Figure 2), and the association with wasp nests was observed significantly more frequently in red‐cheeked cordon‐bleu nests (Figure 3a). While these nesting strategies themselves have already been reported in the past literature (Goodwin, 1982), as far as I know, this study is the first to report on inter‐and intra‐species variation in nesting strategies of cordon‐bleus within the same habitat (Figures 2 and 3). Although my preliminary observations are not conclusive about the costs and benefits of these nesting strategies, my findings suggest that these variations in nesting strategies might be a factor that enables these two species to breed sympatrically. Here, I will discuss the possible functions of the nesting strategies and how to test these in future research.

A typical benefit of association with wasp nests is predator avoidance (Joyce, 1993; Quinn & Ueta, 2008). A past empirical study of red‐cheeked cordon‐bleus has shown that nesting near a wasp nest decreases predation risk and contributes to increasing reproductive success compared to without a wasp nest (Beier & Tungbani, 2006). Nesting associated with other species could also be a risk, such as being attacked by the species (Quinn & Ueta, 2008). However, despite nesting in adjacent areas (Figure 2a,c,d), the cordon‐bleus and wasp are tolerant and do not interfere with each other (Beier & Tungbani, 2006; Ota personal observation). Considering that there were fewer wasp nests around the nests of blue‐capped cordon‐bleus, red‐cheeked cordon‐bleus are likely to have a preference for nesting near wasp nests rather than coincidentally overlapping the nest sites. The presence of wasp nests can be an important cue for nest‐site characteristics of cordon‐bleus because wasps are known to start nest building 1 month earlier than the breeding season of cordon‐bleus (Beier & Tungbani, 2006). This is also supported by the fact that wasp nests require several months to build while cordon‐bleu build nests for around 2 weeks (Beier & Tungbani, 2006; van Someren, 1956).

While I observed that both blue‐capped and red‐cheeked cordon‐bleus took over weaver nests for breeding, only one red‐cheeked cordon‐bleu nest was a weaver nest (Figure 3b). Nest building is energetically costly and time‐consuming (Mainwaring & Hartley, 2013), thus using old weaver nests would significantly save the energy for nest building. Nevertheless, the majority of cordon‐bleus (>80%, see results) built their own nests (Figure 3b). This might be because the number of weaver nests is limited and finding available weaver nests is competitive and time‐consuming. Alternatively, predation risk might be different in the two nest types, which suggest trade‐off between predation risk and energetic costs.

There were no species differences in nest height or nest plant between the two species. I never observed both species of cordon‐bleus nesting in the same tree, suggesting there would be little reason to adjust nest height to reduce nest‐site competition in the same tree. However, since I started to measure nest height in the middle of the study (see Section 2.2) and the sample size is limited, the data could bias the comparison if, for example, cordon‐bleus nested at different heights on different plants and/or earlier in the season (e.g., Ludvig et al., 1995). Thus comparisons should be treated with caution, and I recommend further exploration on nest height differences.

My preliminary observations raise new research questions regarding nest‐site characteristics and nesting behaviors of cordon‐bleus. For example, if nesting near a wasp nest is advantageous for the breeding of red‐cheeked cordon‐bleus, it is puzzling why blue‐capped cordon‐bleus can inhabit sympatrically without a wasp nest. One possible reason is that blue‐capped cordon‐bleus may compensate for any increase in reproductive success they might gain by nesting near a wasp nest by adopting other strategies not detected in the current study. For instance, nest structure and components might vary between blue‐capped and red‐cheeked cordon‐bleus, even if the nests were visually similar (Figure 2a,d). Birds are known to use several types of nest materials for parasite control (Knutie et al., 2014), predator avoidance (Schuetz, 2005), and thermal benefits (Møller, 1991), and the composition is often species specific (Biddle et al., 2018). Another possible reason is that using wasp nests may not contribute much to reproductive success in blue‐capped cordon‐bleu habitat due to some environmental conditions such as predator populations. Blue‐capped cordon‐bleus usually share the habitat with red‐cheeked cordon‐blues, but red‐cheeked cordon‐bleus have broader habitat than blue‐capped cordon‐bleus (Billerman et al., 2020; Goodwin, 1982; Hockey et al., 2005). Using wasp nests may be an effective strategy in other areas dominated by red‐cheeked cordon‐bleus (Beier & Tungbani, 2006). In order to test this, the reproductive consequence of sympatric cordon‐bleus and the effects of nesting strategies should be investigated. It should also be noted that my finding might be area‐specific rather than species‐specific. Environmental differences can cause the inter‐and intra‐species variation in the nest material choice, choosiness, and behavioral plasticity (e.g., Okano et al., 2011). It would be interesting to examine whether cordon‐bleu nesting strategies vary in different regions with different environmental conditions and populations. Such approaches would deepen our understanding of how sympatry and inter‐species variation in nesting strategies evolved and maintained.

## AUTHOR CONTRIBUTIONS

**Nao Ota:** Conceptualization (lead); data curation (lead); formal analysis (lead); funding acquisition (lead); investigation (lead); methodology (lead); project administration (lead); resources (lead); supervision (lead); validation (lead); visualization (lead); writing – original draft (lead); writing – review and editing (lead).

## CONFLICT OF INTEREST

I declare I have no competing interests.

## FUNDING INFORMATION

The research was financially supported by the National Geographic Society (grant number: EC‐KOR‐44731R‐18), the Kawai Foundation for Sound Technology & Music, and the Japan Society for the Promotion of Science (JSPS Overseas Research Fellowships).

## Supporting information


VideoS1

AppendixS1

## Data Availability

The all data for this study are available in the supporting information (Appendix S1).
