# Supplementary material for: A comparison of nest‐site characteristics for two sympatric Estrildid finches (Uraeginthus spp.) in Tanzania
Source: Ecol Evol. 2022 Oct 8;12(10):e9398. doi: 10.1002/ece3.9398 (PMC9547246; doi:10.1002/ece3.9398)
Supplement: Supplementary file 2 — AppendixS1 [file ECE3-12-e9398-s002.docx]

**Supporting information**

Original paper for *Ecology and Evolution*, *Nature Notes*

**Nesting strategy and the niche partitioning in two socially monogamous songbirds**

Nao Ota

**Appendix S1.**

The information of blue-capped and red-cheeked cordon-bleus’ nests that I recorded and analyzed for this study.

| Species | Place | Nest type | Latitude | Longitude | Wasp nest | Height (cm) | Tree type |
| --- | --- | --- | --- | --- | --- | --- | --- |
| Blue-capped cordon-bleu | Chimala | own | -8.731081188 | 34.08189537 | 0 | 366 | acacia |
| Blue-capped cordon-bleu | Chimala | own | -8.731103316 | 34.08215873 | 0 | 196 | acacia |
| Blue-capped cordon-bleu | Chimala | weaver | -8.730456275 | 34.08134719 | 0 | 240 | acacia |
| Blue-capped cordon-bleu | Chimala | own | -8.730905629 | 34.0819113 | 0 | 310 | acacia |
| Blue-capped cordon-bleu | Chimala | own | -8.730946868 | 34.08158842 | 0 | 277 | acacia |
| Blue-capped cordon-bleu | Chimala | weaver | -8.73089029 | 34.08124334 | 0 | 128 | acacia |
| Blue-capped cordon-bleu | Chimala | weaver | -8.738107905 | 34.0750979 | 0 | NA | acacia |
| Blue-capped cordon-bleu | Chimala | own | -8.739692965 | 34.07550585 | 0 | 214 | acacia |
| Blue-capped cordon-bleu | Chimala | own | -8.7398083 | 34.07551532 | 0 | NA | acacia |
| Blue-capped cordon-bleu | Chimala | own | -8.739664382 | 34.07534131 | 0 | 386 | acacia |
| Blue-capped cordon-bleu | Chimala | weaver | -8.733695126 | 34.08124317 | 0 | NA | acacia |
| Blue-capped cordon-bleu | Chimala | own | -8.734590062 | 34.08171365 | 0 | NA | acacia |
| Blue-capped cordon-bleu | Chimala | weaver | -8.732035677 | 34.08140536 | 0 | 240 | acacia |
| Blue-capped cordon-bleu | Chimala | own | -8.72968866 | 34.07621487 | 0 | 250 | acacia |
| Blue-capped cordon-bleu | Chimala | own | -8.731111195 | 34.07239758 | 0 | NA | acacia |
| Blue-capped cordon-bleu | Chimala | own | -8.72838825 | 34.07211361 | 0 | 205 | acacia |
| Blue-capped cordon-bleu | Chimala | own | -8.727868488 | 34.07329881 | 1 | 390 | acacia |
| Blue-capped cordon-bleu | Chimala | weaver | -8.729249113 | 34.07269439 | 0 | NA | acacia |
| Blue-capped cordon-bleu | Chimala | own | -8.732123938 | 34.08132381 | 1 | 300 | acacia |
| Blue-capped cordon-bleu | Chimala | own | -8.730910826 | 34.0818797 | 0 | 261 | acacia |
| Blue-capped cordon-bleu | Chimala | own | -8.730885554 | 34.0818512 | 0 | NA | acacia |
| Blue-capped cordon-bleu | Chimala | own | -8.730932116 | 34.08054077 | 0 | 150 | shrub |
| Blue-capped cordon-bleu | Chimala | own | -8.730717707 | 34.08033935 | 0 | NA | acacia |
| Blue-capped cordon-bleu | Chimala | own | -8.730816194 | 34.08021178 | 0 | NA | acacia |
| Blue-capped cordon-bleu | Chimala | own | -8.730889158 | 34.08193032 | 0 | NA | acacia |
| Blue-capped cordon-bleu | Chimala | weaver | -8.730344963 | 34.07997927 | 0 | NA | acacia |
| Blue-capped cordon-bleu | Chimala | own | -8.730376898 | 34.07922138 | 0 | NA | acacia |
| Blue-capped cordon-bleu | Chimala | own | -8.731192332 | 34.08062962 | 0 | NA | acacia |
| Blue-capped cordon-bleu | Chimala | own | -8.730487037 | 34.08213082 | 0 | NA | acacia |
| Blue-capped cordon-bleu | Chimala | weaver | -8.730742056 | 34.08086532 | 0 | NA | acacia |
| Blue-capped cordon-bleu | Chimala | own | -8.730919333 | 34.08091024 | 0 | 250 | acacia |
| Blue-capped cordon-bleu | Chimala | own | -8.730913214 | 34.08102248 | 0 | 168 | tree |
| Blue-capped cordon-bleu | Chimala | own | -8.731124606 | 34.08054848 | 0 | 276 | acacia |
| Blue-capped cordon-bleu | Chimala | weaver | -8.730744277 | 34.08089155 | 0 | 223 | acacia |
| Blue-capped cordon-bleu | Chimala | own | -8.730737153 | 34.08017591 | 0 | 195 | acacia |
| Blue-capped cordon-bleu | Chimala | own | -8.730750564 | 34.08016811 | 0 | 273 | acacia |
| Blue-capped cordon-bleu | Chimala | weaver | -8.731406573 | 34.08184382 | 0 | NA | acacia |
| Blue-capped cordon-bleu | Chimala | own | -8.730731285 | 34.08066675 | 0 | 400 | tree |
| Blue-capped cordon-bleu | Chimala | own | -8.732019374 | 34.08134803 | 0 | 330 | acacia |
| Blue-capped cordon-bleu | Chimala | own | -8.732019374 | 34.08134803 | 0 | NA | acacia |
| Blue-capped cordon-bleu | Chimala | own | -8.739664382 | 34.07534131 | 0 | 324 | acacia |
| Red-cheeked cordon-bleu | Chimala | own | -8.733873619 | 34.07996669 | 1 | 153 | tree |
| Red-cheeked cordon-bleu | Chimala | own | -8.730281806 | 34.07988564 | 1 | 160 | acacia |
| Red-cheeked cordon-bleu | Chimala | own | -8.732042718 | 34.08133118 | 1 | 300 | acacia |
| Red-cheeked cordon-bleu | Chimala | own | -8.730097697 | 34.07783358 | 1 | 342 | acacia |
| Red-cheeked cordon-bleu | Chimala | own | -8.730097697 | 34.07783358 | 1 | 250 | acacia |
| Red-cheeked cordon-bleu | Chimala | own | -8.818406705 | 34.03123531 | 1 | 250 | acacia |
| Red-cheeked cordon-bleu | Chimala | own | -8.818067405 | 34.03058798 | 0 | NA | acacia |
| Blue-capped cordon-bleu | Iringa | own | -7.817260455 | 35.09352185 | 0 | 188 | acacia |
| Blue-capped cordon-bleu | Iringa | own | -7.818268505 | 35.09288164 | 0 | 302 | shrub |
| Red-cheeked cordon-bleu | Iringa | own | -7.805491928 | 35.75467782 | 1 | 330 | acacia |
| Red-cheeked cordon-bleu | Iringa | own | -7.79839078 | 35.76774403 | 0 | 200 | tree |
| Red-cheeked cordon-bleu | Iringa | own | -7.822015048 | 35.08509242 | 0 | NA | acacia |
| Red-cheeked cordon-bleu | Iringa | own | -7.819411419 | 35.09189609 | 1 | 370 | acacia |
| Red-cheeked cordon-bleu | Iringa | own | -7.817978868 | 35.09687168 | 1 | 340 | acacia |
| Red-cheeked cordon-bleu | Iringa | weaver | -7.817913531 | 35.0946819 | 1 | 250 | acacia |
| Red-cheeked cordon-bleu | Iringa | own | -7.814911804 | 35.09358522 | 1 | 160 | acacia |
| Red-cheeked cordon-bleu | Iringa | own | -7.814771826 | 35.09378856 | 1 | 277 | acacia |
